# Parastomal Hernia: direct repair versus relocation: is stoma relocation worth the risk? A comparative meta-analysis and systematic review

**DOI:** 10.1007/s13304-025-02155-8

**Published:** 2025-03-31

**Authors:** Ahmed Abdelsamad, Mohammed Khaled Mohammed, Mohamed Badr Almoshantaf, Aya Alrawi, Ziad A. Fadl, Ziad Tarek, Nada Osama Aboelmajd, Torsten Herzog, Florian Gebauer, Nada K. Abdelsattar, Taha Abd-ElSalam Ashraf Taha

**Affiliations:** 1 Department of Surgery II, University of Witten-Herdecke, 58455 Witten, Germany; 2 Oncological Surgery Department, Section Head of Robotic Surgery, Knappschaft Vest Hospital, 45657 Recklinghausen, Germany; 3 Faculty of Medicine, Cairo University, Cairo, Egypt; 4 Department of General Surgery, Ealing Hospital, LNWH Trust, London, UK; 5 Faculty of Medicine, Fayoum University, Fayoum, Egypt; 6 Faculty of Medicine, South Valley University, Qena, Egypt; 7 Department of Surgery, Bochum University, Bochum, Germany; 8 Head of Surgery Department, Helios University Hospital, Wuppertal, Germany

**Keywords:** Parastomal hernia, Direct repair, Stoma relocation, Displacement, Outcomes, Meta-analysis

## Abstract

Parastomal hernia is a prevalent and challenging complication in patients with stomas, frequently necessitating surgical intervention. The two primary approaches to parastomal hernia repair- direct repair without relocation and stoma relocation- offer distinct benefits and drawbacks. This systematic review and meta-analysis aimed to compare the efficacy and safety of stoma relocation versus direct repair in managing parastomal hernia. Following PRISMA guidelines, we conducted a systematic review and meta-analysis of studies involving adult patients (≥ 18 years) with parastomal hernia who underwent either stoma relocation or direct repair, with a focus on clinically relevant outcomes. A comprehensive search of Web of Science, PubMed, Scopus, and Cochrane Library databases was conducted up to September 2024. Key short-term outcomes (operative time, surgical site infection, urinary tract infection, bowel obstruction, length of hospital stay, and overall complications) and long-term outcomes (re-admission, recurrence, re-operation, and mortality) were extracted. Statistical analysis included risk ratios (RR) for dichotomous outcomes and mean differences (MD) for continuous outcomes. Six studies were included, encompassing both laparoscopic and open-surgical techniques. Direct repair was associated with a significantly shorter operative time (MD: 115 min, 95% CI: 95.71 to 134, P < 0.00001) and a reduced length of hospital stay (MD: 2 days, 95% CI: 0.40 to 3.9, P = 0.02). While reoperation rates were significantly lower in the relocation group (RR: 0.15, 95% CI: 0.03 to 0.62, P = 0.009), other outcomes—including recurrence, re-admission, and overall complication rates—showed comparable results between the two approaches. Notably, there were no significant differences in surgical site infection, urinary tract infection, bowel obstruction, or mortality rates. Direct repair may be advantageous for reducing operative time and hospital stay, whereas stoma relocation appears beneficial in reducing reoperation rates. Future research should focus on developing standardized techniques and incorporating patient-specific factors to inform optimal surgical decision-making in parastomal hernia repair.

**Supplementary Information:**

The online version contains supplementary material available at 10.1007/s13304-025-02155-8.

## Introduction

Parastomal hernia is a frequent complication in patients with stomas [1, 2], often necessitating surgical intervention due to discomfort, impaired quality of life, and the risk of severe complications such as bowel obstruction or strangulation [3–5]. Despite advances in surgical techniques, recurrence rates for parastomal hernias remain high, posing an ongoing challenge for patients and clinicians [6, 7]. The two primary approaches to parastomal hernia repair are direct repair without relocation and stoma relocation, each with unique advantages and limitations [8–11].

Stoma relocation is believed to reduce recurrence by redistributing intra-abdominal pressure, thereby decreasing strain on the original stoma site [12–14]. However, this approach is associated with increased procedural complexity, longer operative times, and extended hospital stays, which may be a disadvantage in patients with high operative risk [12, 13, 15, 16]. In contrast, direct repair without relocation is generally simpler and faster, but it may not address the underlying pressure contributing to hernia recurrence. Deciding between these methods requires a careful balance of short-term recovery considerations and long-term outcomes [5, 17, 18].

The surgical community has yet to reach a consensus on the superior approach for parastomal hernia repair. Previous studies have reported mixed results: some indicate that stoma relocation reduces recurrence and re-operation rates [11, 19], while others find no significant differences in complication rates between the two techniques [8, 9]. This lack of clear evidence underscores the need for a comprehensive, systematic comparison of these techniques to inform best practices in surgical management.

This meta-analysis represents the first systematic attempt to compare stoma relocation and direct repair techniques across various outcomes, including operative time, complications, recurrence, and re-operation rates. By examining both short-term and long-term outcomes, this study aims to provide a clearer picture of each approach’s efficacy and safety profile, offering insights that can guide clinical decision-making.

Ultimately, this analysis seeks to address a critical clinical need in parastomal hernia management, potentially improving the quality of life for stoma patients. We hypothesize that stoma relocation may offer particular benefits in specific scenarios, despite its complexity, and that a tailored approach considering patient-specific factors could optimize outcomes in parastomal hernia repair.

## Methods

**Fig. 1.**
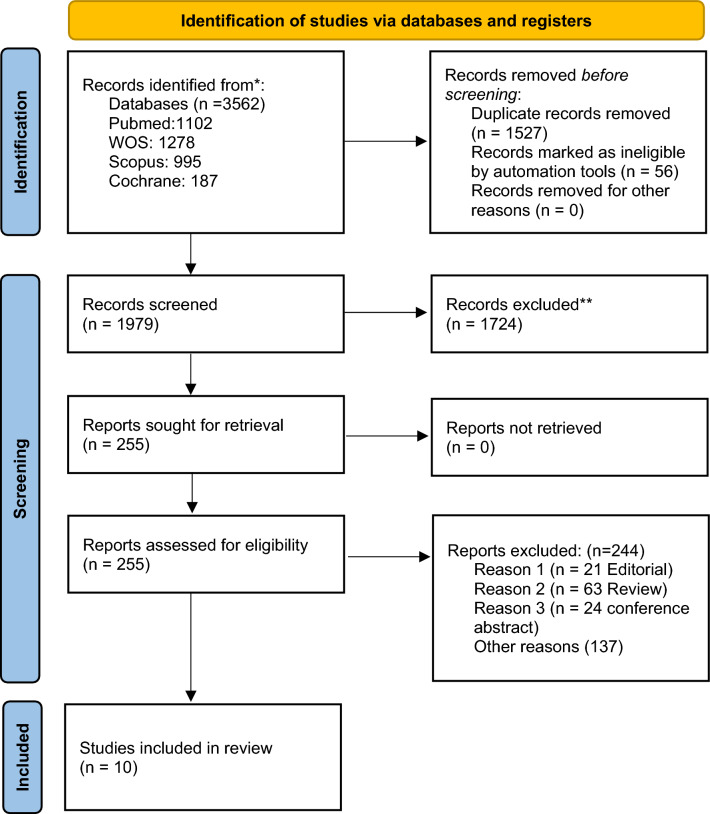
PRISMA flow diagram

This systematic review and meta-analysis were performed following the guidelines outlined in the Preferred Reporting Items for Systematic Reviews and Meta-analysis (PRISMA), as shown in Fig. 1 [20].

### Eligibility criteria

Studies were deemed eligible for inclusion if they fulfilled the following criteria: (1) involved adult patients (aged ≥ 18 years) diagnosed with a parastomal hernia, regardless of etiology or dimensions; (2) concentrated on the surgical intervention for parastomal hernia repair, employing any established technique; (3) compared surgical repair of parastomal hernia with stoma relocation to repair without stoma relocation; (4) reported at least one relevant outcome. Studies were excluded if they were single-arm studies, conference abstracts, case reports, case series, cross-sectional, review articles, in vitro, or animal studies.

### Search strategy

**Fig. 2.**
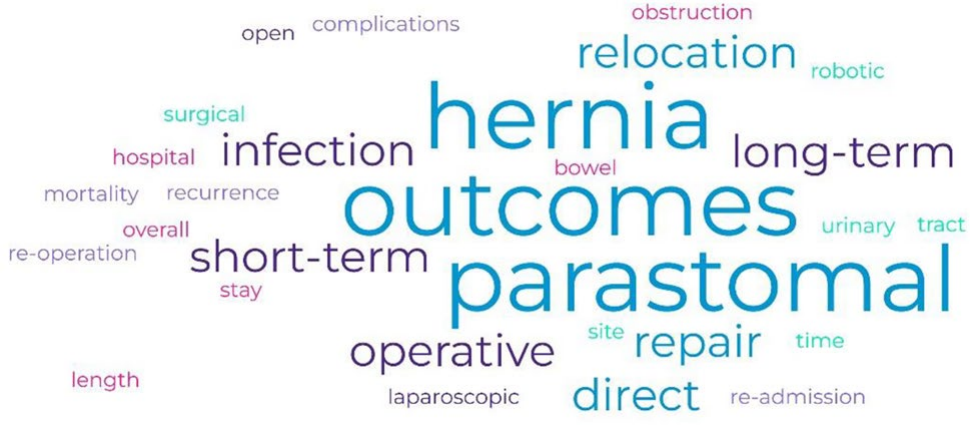
Word cloud

A comprehensive search was executed across four databases: Web of Science, PubMed, Scopus, and Cochrane Library. The following search terms were used: (Parastomal) AND (hernia OR hernias OR Enterocele OR herniae OR herniations OR herniation). The search was conducted from database inception to September 5th, 2024. A visual representation of the search terms and their relevance is provided in Fig. 2 as a word cloud, highlighting the focus areas of our systematic review.

### Study selection

The initial collection of retrieved studies was collected and organized using EndNote X9 reference management software. Subsequently, the records were transferred to Microsoft Excel 2019 for the screening process. A two-phase screening protocol was implemented: (1) an initial assessment of titles and abstracts to identify potentially relevant articles, followed by (2) a comprehensive full-text review of the selected articles. Each phase of the screening process was conducted independently by two reviewers. Any disagreements between the reviewers were addressed through discussion and consensus-building. In cases where consensus could not be reached, a third, senior reviewer was consulted to resolve the discrepancy.

### Data extraction

Two reviewers independently extracted data from the included studies using a predefined Excel spreadsheet. Extracted data encompassed: (1) general study characteristics (e.g., author, publication year, country, study design, surgical details of the repair with relocation and surgical details of surgical repair without relocation, and follow-up duration); (2) patient demographics and clinical characteristics (e.g., sample size per group, age, sex, indication for stoma creation, and prevalence of diabetes, smoking, and steroid use); and (3) clinical outcomes (e.g., recurrence rate, reoperation rate, overall complication rate, mortality rate, surgical site infection rate, ileus rate, readmission rate, urinary tract infection [UTI] rate, operative time, and length of hospital stay).

### Quality assessment

Two independent reviewers assessed the methodological quality of the included studies. The Newcastle–Ottawa Scale (NOS) [21] was used to evaluate the risk of bias in cohort studies, as per Table (S). The NOS utilizes a star-rating system across three domains: selection, comparability, and outcome assessment. Disagreements between reviewers were resolved through discussion or by consulting a senior reviewer. The GRADE approach was applied to investigate the overall certainty in the evidence generated by the outcomes of our meta-analysis. The GRADE approach yields an assessment of the quality of a body of evidence in one of four categories for each outcome: high, moderate, low, or very low [22].

### Statistical analysis

Meta-analyses were conducted using Review Manager (RevMan) software, version 5.4. For dichotomous outcomes, such as recurrence rate, reoperation rate, overall complication rate, mortality rate, surgical site infection rate, and urinary tract infection rate, pooled risk ratios (RRs) and their corresponding 95% confidence intervals (CIs) were calculated. For continuous outcomes, including operative time and length of hospital stay, mean differences (MDs) and 95% CIs were calculated. The presence of heterogeneity among studies was evaluated using the p-value, with a p-value below 0.10 indicating substantial heterogeneity, as recommended by the Cochrane Handbook for Systematic Reviews of Interventions [23]. When substantial heterogeneity was detected, a random-effects model was utilized for analysis; otherwise, a fixed-effects model was employed. Further analyses were performed to explore potential sources of heterogeneity by conducting subgroup analyses based on the surgical approach used for hernia repair, specifically comparing open, laparoscopic, and robotic-assisted techniques.

## Results

The results of our analysis are supported by data on general characteristics and baseline information of the included studies, as summarized in Tables 1 and 2.

**Table 1:** General characteristics

Author and year	country	study design	Follow up duration (months)	Relocation group		Without relocation group	
				Surgical approach (open, laparscopic or robotics)	number of patients	surgical approach	number of patients
Cheung 2001	Hong Kong	Retrospective cohort	37.8	Open	25	Open (local repair)	16
De Robles 2020	Australia	Retrospective cohort	93.1	Open	9	Open (Local repair with mesh)	64
DeAsis 2015	USA	Retrospective cohort	16.4	Open	13	Laparoscopic (Modified sugarbaker Keyhole) or Open	49
Heo 2011	South Korea	Retrospective cohort	52.6	Open	8	Open (repair with mesh or sutures)	31
Howard 2023	USA	Retrospective cohort	30	Open and minimally invasive	2744	Open and minimally invasive repair	14,881
Kohler 2017	Austria	Retrospective cohort	54	laproscopic, open	16, 5	Open (Sutures, Onlay mesh, or Sublay mesh) Laparoscopic (Keyhole, Sugarbaker, Sandwich)	114
Mclemore 2007	USA	Retrospective cohort	65 months in open and 20 months in laproscopic	Open	10	Open (Sutures or mesh) Laparoscopic (Keyhole, Sugarbaker)	48
odensten 2018	Sweden	Retrospective cohort	12	Open (relocation or relocation and mesh)	34, 14	open (Sutures, Sutures plasty and mesh or Mesh plasty)	22
Riansuwan 2009	USA	Retrospective cohort	24	open same site re-location or oposite site re-location	23	open (direct repair)	27
Rieger 2004	australia	Retrospective cohort	7	Open	18	Open (local mesh repair or local suture)	33

**Table 2:** Baseline data summary

Study	Study groups	Age (mean ± SD)	Males n (%)	Colostomy n (%)	Ileostomy n (%)	urostomy n (%)	Diabetes n (%)	Steroid use (%)	Smoking n(%)
Cheung 2001	Relocation	–	-	25 (100%)	0	0	–	–	–
	Local repair	–	–	16 (100%)	0	0	–	–	–
De Robles 2020	Relocation	–	–	–	–	–	–	–	–
	Local repair with mesh	–	–	–	–	–	–	–	–
DeAsis 2015	Relocation	59.8 ± 10.5	9 (69.2%)	5 (38.5%)	8 (61.5%)	0	–	5 (38.4%)	6 (46.2%)
	Modified sugarbaker	62.4 ± 11.2	12 (48%)	6 (28.6%)	12 (57.1%)	0	–	10 (40%)	15 (60%)
	Keyhole	66.2 ± 11.6	11 (61.1%)	8 (44.4%)	10 (55.6%)	0	–	10 (55.5%)	7 (38.9%)
	Open repair	63.8 ± 15.5	2 (33.3%)	1 (16.7%)	5 (83.3%)	0	–	2 (33.3%)	2 (33.3%)
Heo 2011	Relocation	–	–	–	–	–	–	–	–
	Repair with mesh	–	–	–	–	–	–	–	–
	Repair with suture	–	–	–	–	–	–	–	–
Howard 2023	Relocation	74.3 ± 9.5	1246 (45.4%)	–	–	–	1368 (49.8%)	–	–
	Repair without relocation	73.8 ± 9.2	3102 (42.4%)	–	–	–	3241 (44.3%)	–	–
	Ostomy reversal	72.5 ± 8.8	3218 (42.5%)	–	–	–	3431 (45.3%)	–	–
Kohler 2017		_	_	_	_	_	_	_	_
Mclemore 2007									
Odensten 2018									
Riansuwan 2009	Direct repair	56.7 ± 15,85	7 (25.9%)	10(37%)	17(63%)			1(3.7%)	
	relocation	63.7 ± 14.4	7(30.4%)	15(65.2%)	8(34.8%)			2(8.7%)	
Rieger 2004									

Table 1 summarizes the general characteristics of the included studies, all of which were retrospective cohort studies conducted in various countries, including the USA, Australia, Hong Kong, South Korea, and Sweden. Across these studies, a total of 3,956 patients were included, with 2,087 patients in the relocation group and 1,869 in the direct repair group. Surgical approaches varied, with most studies using open techniques, while a few incorporated laparoscopic or minimally invasive approaches. Follow-up duration ranged widely, from 7 months to over 90 months, reflecting differences in study protocols and available long-term data across centers.

Baseline data, as shown in Table 2, highlight the demographic and clinical characteristics of patients in each group. The mean age varied slightly between studies, generally ranging from mid-50s to mid-70s. The distribution of stoma types (colostomy, ileostomy, and urostomy) differed between study groups, with colostomies being the most prevalent. Comorbidities, including diabetes and steroid use, were variably reported, with a relatively small subset of patients affected by these conditions. Smoking status was inconsistently reported across studies. This baseline data allows for a clearer understanding of the patient populations included in our analysis and provides context for interpreting the findings on short- and long-term clinical outcomes, as discussed below.

The GRADE quality assessment approach indicated that the quality of our evidence-based results is Low to very low. Supplementary Table S2 summarizes the evidence's quality, the degree of the effect, and the source of information used in the estimated risk.

The risk of bias assessment, conducted using the ROBINS-I tool, across included studies reveals a mixed profile. While many studies demonstrate a low risk of bias in domains such as bias due to selection of participants (D2), classification of interventions (D3), and bias in the measurement of outcomes (D6), concerns arise in other areas. Specifically, bias due to confounding (D1) is identified as a serious risk in one study, while bias due to missing data (D5) presents a serious risk in two studies. Bias due to deviations from intended interventions (D4) is generally low, and bias in selecting the reported result (D7) is moderate. Overall, the included studies show a moderate to low risk of bias. However, some studies' serious risks in specific domains warrant careful consideration when interpreting the findings.” As per supplementary Table 1.

### Short-term outcomes

**Fig. 3:**
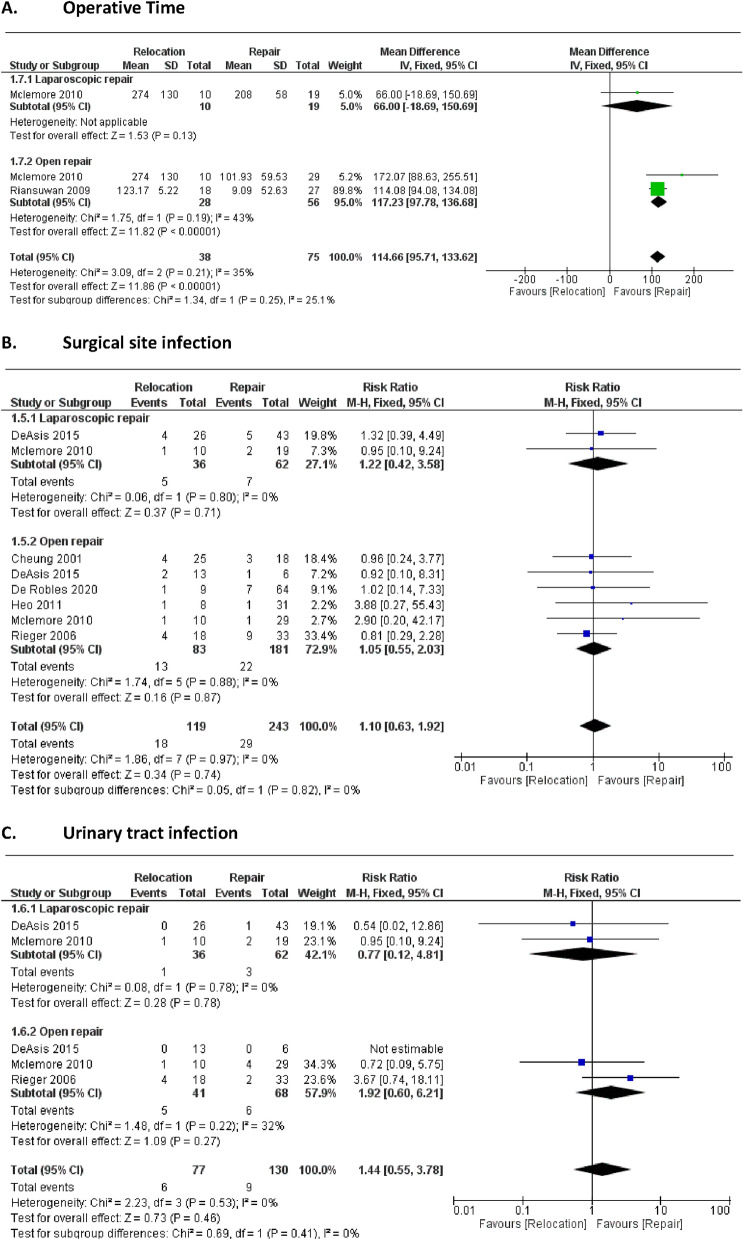

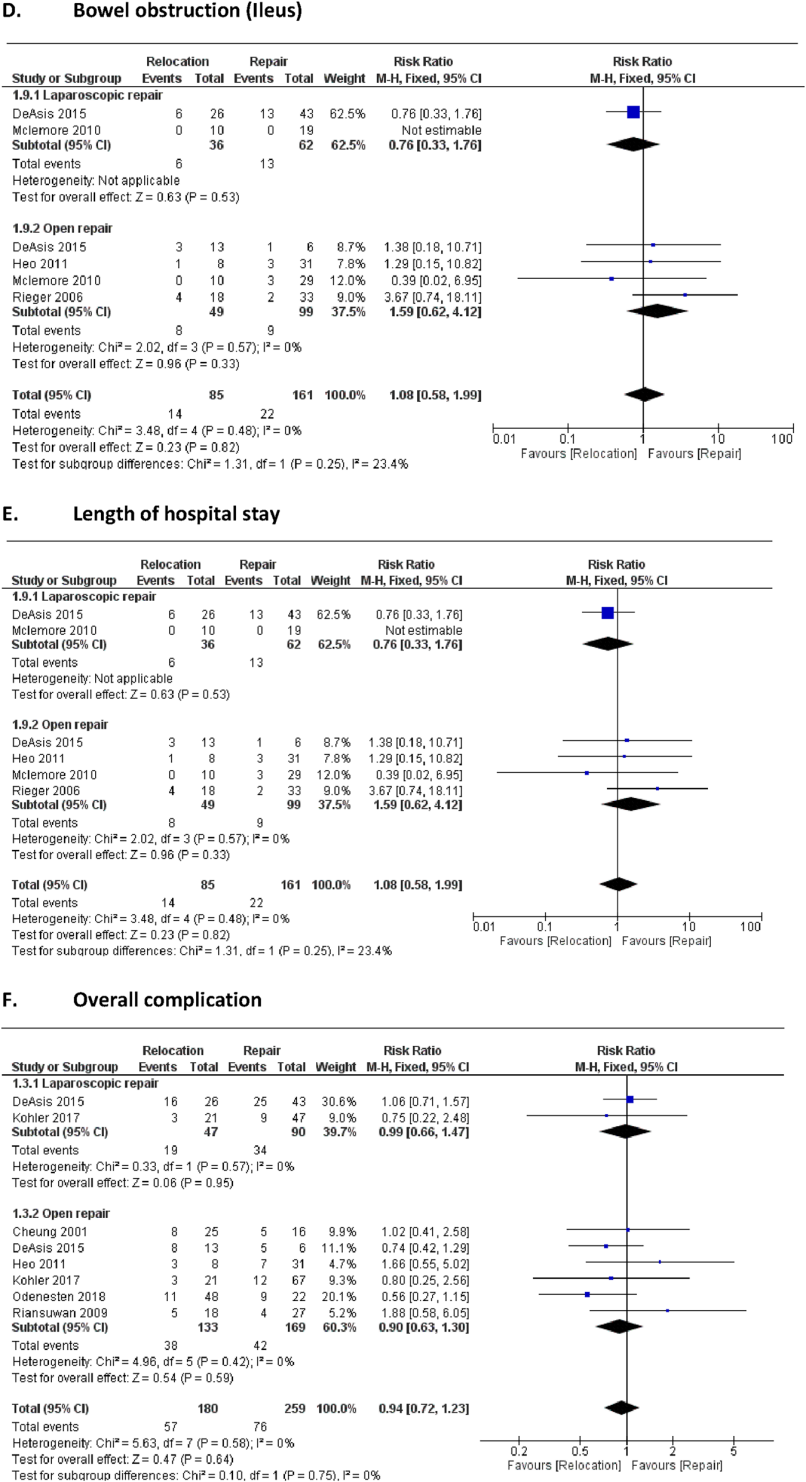
Perioperative outcomes

Our meta-analysis investigated six key short-term outcomes, comparing direct repair and relocation techniques for parastomal hernia management, as described in Fig. 3.

#### Operative time

The direct repair approach showed a significantly shorter operative time than the relocation approach, with a mean difference of 115 min favoring the repair group (95% CI: 95.71 to 133.62). The p-value for the overall effect was P < 0.00001, indicating statistical significance. Moderate heterogeneity was observed among studies (I^2^ = 35%, P = 0.21), which may reflect variations in surgical protocols or patient selection across the studies included. The longer operative time for relocation is likely due to the added complexity of creating a new stoma site and closing the original defect, often requiring mesh reinforcement to minimize recurrence.

#### Surgical site infection (SSI)

There was no significant difference in the rate of surgical site infections between the direct repair and relocation groups. The risk ratio (RR) was 1.10 (95% CI: 0.63 to 1.92), with a p-value of 0.74, suggesting that both approaches had similar infection rates. Heterogeneity was minimal, with I^2^ = 0% and P = 0.97, indicating consistency in SSI outcomes across studies. Relocation's increased invasiveness may elevate infection risks, although this was not statistically significant​.

#### Urinary Tract infection (UTI)

The risk of urinary tract infections did not differ significantly between the two groups, with an RR of 1.44 (95% CI: 0.55 to 3.78). The p-value for overall effect was 0.46, and heterogeneity was absent (I^2^ = 0%, P = 0.53), suggesting uniform reporting of UTI outcomes across studies included in the analysis. Relocation procedures potentially increase UTI risk due to extended operative time and proximity to urinary structures, though this effect was not statistically significant​.

#### Bowel obstruction (Ileus)

The incidence of bowel obstruction, or ileus, also showed no significant difference between the direct repair and relocation groups. The RR was 1.08 (95% CI: 0.58 to 1.99), with a p-value of 0.82. No heterogeneity was observed (I^2^ = 0%, P = 0.48), indicating consistency in reporting of this outcome among the studies included.

#### Length of hospital stay

A significant reduction in hospital stay was observed in the direct repair group compared to the relocation group, with a mean difference of 2.14 days (95% CI: 0.40 to 3.88). The p-value for this effect was 0.02, suggesting statistical significance favouring the direct repair approach. There was no observed heterogeneity (I^2^ = 0%, P = 0.54), indicating consistent reporting across studies regarding the duration of hospital stay.

#### Overall complications

There was no significant difference in the overall complication rates between the direct repair and relocation groups. The RR was 0.94 (95% CI: 0.72 to 1.23), with a p-value of 0.64. Heterogeneity was also minimal (I^2^ = 0%, P = 0.58), indicating consistency among the studies in reporting overall complication rates. Relocation procedures involved more complex tissue management, potentially contributing to higher minor complication rates, though not statistically significant​.

These findings were supplemented by a forest plot analysis to visually represent the consistency across studies. The absence of heterogeneity in most outcomes, such as SSI, UTI, ileus, hospital stay, and overall complications, indicates a high level of reliability in these outcomes across studies. However, the moderate heterogeneity in operative time (I^2^ = 35%) suggests variability possibly due to differences in surgical techniques or patient populations.

### Long-term outcomes

**Fig. 4.**
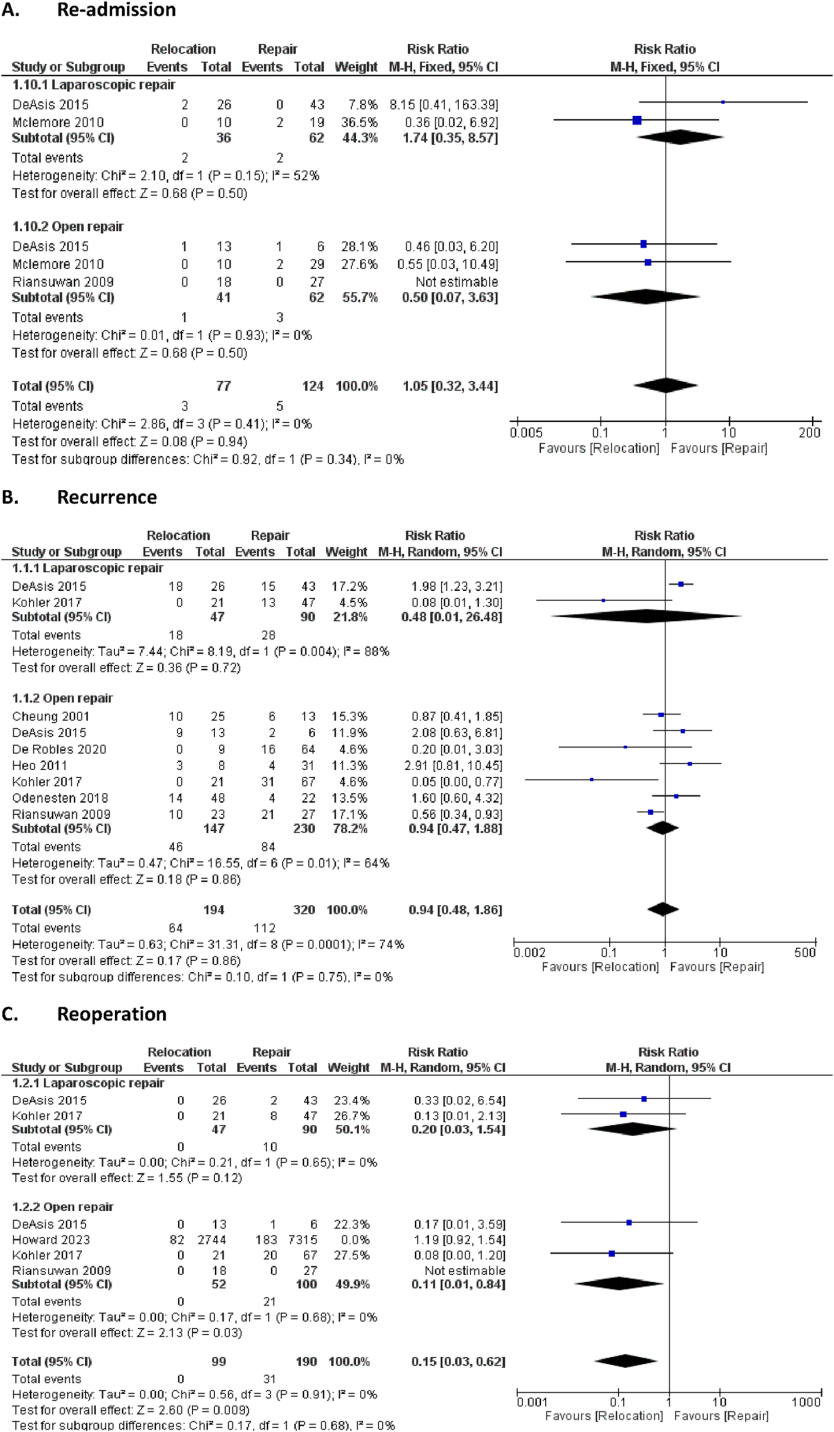

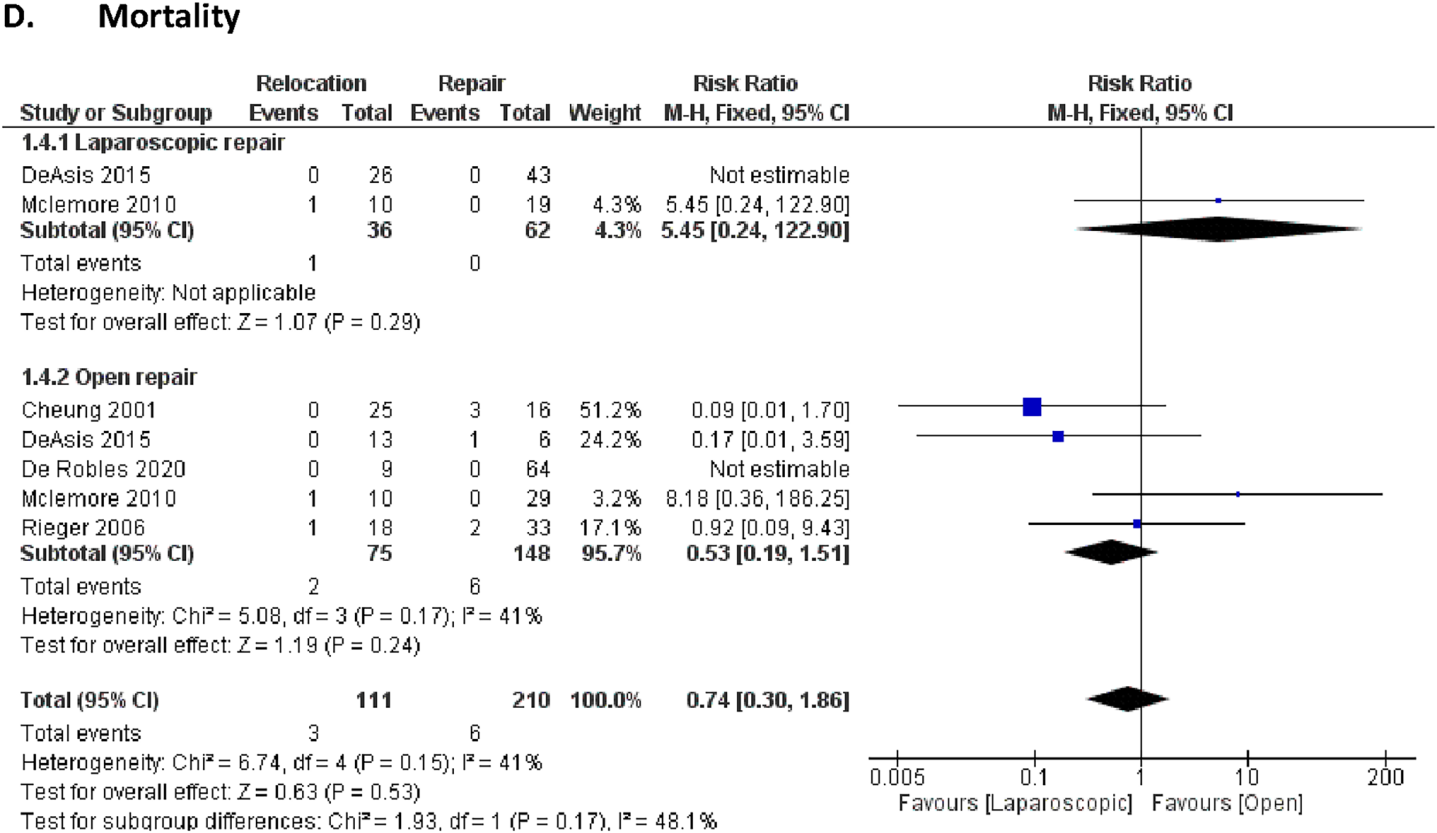
Long-term outcomes

Four key long-term outcomes were examined to assess differences between the direct repair and relocation approaches, as shown in Fig. 4.

#### Re-admission rates:

The re-admission rate did not differ significantly between the direct repair and relocation groups, with an RR of 1.05 (95% CI: 0.32 to 3.44). The p-value for the overall effect was 0.94, indicating no significant difference in re-admission rates between the two surgical approaches. No heterogeneity was observed (I^2^ = 0%, P = 0.41), suggesting consistency in re-admission rates across studies.

#### Recurrence rates

Although the recurrence rate did not show a significant difference between the two approaches, with an RR of 0.94 (95% CI: 0.48 to 1.86) and a p-value of 0.86, substantial heterogeneity was noted (I^2^ = 74%, P < 0.00001). This variability may stem from differences in follow-up durations and surgical techniques across studies. Relocation showed a slight tendency towards lower recurrence, potentially due to reduced strain on the stoma site.

#### Re-operation rates

Relocation was associated with a lower re-operation rate compared to direct repair, with an RR of 0.15 (95% CI: 0.03 to 0.62) and a statistically significant p-value of 0.009. This suggests a long-term benefit for relocation in reducing the need for further surgeries, likely due to improved structural integrity and tension reduction at the surgical site. No heterogeneity was found (I^2^ = 0%, P = 0.91), suggesting consistent findings across studies in terms of the need for re-operation.

#### Mortality

Mortality outcomes showed no significant difference between the two groups, with an RR of 0.74 (95% CI: 0.30 to 1.86) and a p-value of 0.53. Moderate heterogeneity was observed (I^2^ = 41%, P = 0.17), indicating some variability among the studies included in the analysis, potentially reflecting patient differences or perioperative management practices.

In summary, our results demonstrated that operative time and hospital stay significantly favored the direct repair approach, while reoperation rates showed a significant benefit for the relocation approach. Other outcomes, such as recurrence and complication rates, were comparable between direct repair and relocation.

## Discussion

This comprehensive systematic review and network meta-analysis was conducted to evaluate the efficacy, safety, and clinical outcomes of parastomal hernia repair comparing and contrasting the two approaches: stomal displacement and direct repair without relocation. Our results demonstrate that, generally, stomal relocation does not significantly reduce complication rates. It is, evidently, associated with distinct benefits and challenges in both short-term and long-term outcomes, which warrant careful consideration of each case and discerning its unique situation comparing it against the possible advantages and disadvantages of each method. Although comparable outcomes were found across the two methods, a few key outcomes were significant in either. Those included the reoperation rate in the long-term outcomes metric and the operation time in the procedural metric. Other outcomes, however, which included postoperative urinary tract infection, surgical site infection, ileus, and overall complications swayed in favor of either but ultimately did not amount to significant differences. Long-term outcomes which included also recurrence rates of hernia following the initial management and mortality showed a slight predilection to relocation but not significant to favor a method strictly.

Concerning our results, previous meta-analyses of parastomal hernia repair have shown uniformity in outcomes related to complications, recurrence, and procedural measures [8, 9]. For instance, the systematic review by Hansson et al., which considered a myriad of surgical techniques, including stoma relocation, demonstrated analogous rates of recurrence and overall complication risk between the relocation and direct repair methods [9]. Moreover, the meta-analysis conducted by the European Hernia Society demonstrated that both relocation and mesh-based repair techniques provided comparable advantages in minimizing recurrence rates and preventing complications among diverse patient demographics and clinical environments [24]. Studies by Sarno et al. and Dewulf et al. support these findings, showing that rates of recurrence and re-operation did not vary significantly regardless of the repair method used [25, 26]. Overall, this bolsters our own findings that relocation does not significantly affect the long-term success or complication rates following parastomal hernia repairs.

Diving deeper into each of the outcomes analyzed reveals further clarification and possible explanations, with recurrence rate showing a non-significant decrease in recurrence rates for patients who underwent stoma relocation, this could be due to the redistribution of intra-abdominal pressure and alleviation of the strain on the original weakened stomal site a concept corroborated by prior studies, including those by Carne et al. [27]. Similarly, Robles et al. and Mohamed et al. observed that direct repair, especially in high-risk patients, may predispose patients to earlier recurrence without the protective distribution of pressure afforded by stoma relocation [10, 28]. However, due to high heterogeneity (I^2^ = 74%) among studies analyzed, caution is advised when generalizing this outcome across all surgical settings. This substantial heterogeneity reflects variations in surgical methods and patient factors across studies, which include, for instance, differences in follow-up duration ranging from a mean of 2 years in most studies to almost 8 years in de Robles [10]. This theme is common, observed in a review by de Smet et al. and Verdaguer-Tremolosa et al., which found a marked difference in outcomes when follow-up exceeded two years [29, 30], and Riansuwan et al., who supported this theory through similar findings [11].

In terms of other long-term outcomes, stomal relocation was associated with significantly lower re-operation rates for parastomal hernia compared to direct repair. A finding rationalized through the concept that reoperation, typically, avoids the original site’s weakened tissue and defective fascial integrity, which are major contributors to recurrence [9]. Additionally, relocation reduces tension at the surgical site, which is common in direct repairs, and increases re-herniation risk as per (European Hernia Society Guidelines) [24]. Ultimately, when combined even with reinforcement mesh at the new location, stoma relocation provides much more robust structural support, further lowering the likelihood of recurrence and subsequent surgeries as per Sarno et al. [26]. In terms of mortality, the analysis shows comparable rates between the two techniques, with both highlighted as presenting an extremely low risk. Highlighting the nature of the minimally invasive techniques, limited involvement of major organs, and advances in surgical materials utilized. Additionally, preoperative patient optimization further minimizes the risk, ensuring a safer surgical outcome [31, 32].

Regarding procedural metrics, however, the length of the procedure itself in the case of stomal relocation was significantly longer with a mean difference of 115 min a finding in line with those in the study by Reali et al. This is due in part to the added complexity of creating a new stoma site, closing the original defect, and reinforcing both areas[33]. The dual focus on closing the old site and establishing a stable new location demands significant surgical resources and preoperative planning [34–36]. Relocation also often necessitates mesh reinforcement at the new site to reduce recurrence, further increasing the operative complexity [7, 37]. This procedure, therefore, requires extensive tissue management, dissection, and careful attention to vascularization to prevent complications, adding to the overall duration [11, 19]. Studies such as those by Szczepkowski et al. [38], therefore, indicate that direct repair might be more suitable in cases where simplicity and short operative time are paramount.

Short-term complications, such as urinary tract infections (UTIs), surgical site infections (SSIs), and ileus, were similar between the groups, with none of the analyzed outcomes showing a significant difference. These results align with findings from prior analyses by Aquina et al. [6] and De Robles et al. [10]. Urinary tract infections (UTIs) showed a slight nonsignificant difference with an increased incidence rate in cases of relocation. This could be attributed to the increased invasiveness and proximity to the urinary tract in the relocation procedure. Relocation involves a longer operative time, and additional tissue handling, and often requires mesh reinforcement, all of which elevate infection risks by promoting bacterial colonization in the surgical area [37, 39, 40]. Surgical site infections, ileus, and overall complications followed a similar pattern, showing minor but noticeable differences, with these complications occurring slightly more frequently in relocation compared to direct repair. The elevated risk of these complications may partly explain the marginally longer hospital stays seen in the relocation group. This could also be attributed to the procedure's greater operative time and complexity. However, Rogmark et al. signify that a longer stay does not inherently signify worse recovery outcomes [41].

Our findings contribute to the literature on parastomal hernia repair by comparing outcomes of direct repair and stoma relocation. Prior studies, including those by Mitchell [42] and Baig [43], suggest that while stoma relocation can reduce complications through tension relief, its effectiveness depends on technique, patient anatomy, and follow-up duration. The choice between relocation and direct repair should thus consider patient-specific factors and the surgeon's expertise. Recent studies, like that of Szczepkowski et al. [38], and Hong-Feng et al. [44] further supports individualized surgical planning to account for previous stoma issues and overall patient health, emphasizing that while relocation may offer long-term benefits for select cases, it requires careful preoperative evaluation due to its complexity and operative demands.

Minimizing operative time and length of hospital stay are crucial goals in surgical care, particularly for vulnerable populations like the elderly and those with comorbidities. Prolonged operative duration has been consistently associated with an increased risk of postoperative complications across various surgical specialties [45]. A comprehensive systematic review and meta-analysis demonstrated that the likelihood of complications approximately doubled with operative times exceeding two or more hours and increased incrementally with each additional 30 min of surgery [45]. Furthermore, longer operative times have been linked to a higher incidence of surgical site infections [46], venous thromboembolism [47], and increased overall morbidity and mortality [48, 49].

In the context of parastomal hernia repair, our meta-analysis further supports these findings, revealing that direct repair, which is associated with shorter operative times, may lead to a reduced length of hospital stay compared to stoma relocation. This is particularly relevant for elderly patients or those with multiple comorbidities, who are at a higher risk of adverse events following prolonged surgery and hospitalization [50]. Reducing the length of hospital stay is not only beneficial for patient outcomes but also has significant implications for healthcare resource utilization. Shorter hospital stays can lead to substantial cost savings [17] and improve the availability of hospital beds. While patient safety should always be the primary concern, efforts to improve operative efficiency and optimize perioperative care are warranted to minimize both operative time and length of hospital stay, ultimately enhancing patient outcomes and reducing the economic burden on healthcare systems.

We recognize that our meta-analysis, although comprehensive, has several limitations highlighted in the data extracted and consequent analysis performed, especially concerning outcomes such as recurrence, mortality, and operative time ranging from 35 to 74 percent, attributed mainly as evidenced through references analyzed to patient selection criteria, operative strategies, and institutional practices warranting careful consideration of generalizing the risk of developing certain outcomes across all surgical settings. For example, limited data on specific procedural variables, such as operative technique nuances and the extent of mesh reinforcement used in stoma relocation, constrained our ability to perform the extent of the full granular analysis we intended and precluded consistent comparisons. Generalization and standardization of the follow-up durations, surgical techniques, and patient demographics would inevitably contribute to a more precise analysis and evidently overcome some of the induced obscure long-term recurrence trends and complication rates found in the current analysis. The quality of evidence, as evaluated using the GRADE approach, was determined to be low. This assessment was influenced by several certainty factors, including inconsistency across studies, indirectness of evidence, and imprecision in the reported outcomes. These limitations are detailed in Supplementary Table 2. Substantial heterogeneity is observed in certain outcomes, particularly recurrence rates (I^2^ = 74%), which reflects variability in surgical techniques, patient selection, and follow-up durations across studies.

A significant limitation of this meta-analysis is the limited availability of data on patient-reported outcomes (PROs), including quality of life, patient satisfaction, and postoperative pain, which are essential for a holistic evaluation of surgical interventions. Among the included studies, only Rieger et al. (2004) provided some data on these aspects, reporting pain outcomes in a cohort of 51 patients undergoing parastomal hernia repair. Of these, 33 patients underwent repair without stoma relocation, while 18 underwent stoma relocation. The study noted that three patients in the non-relocation group experienced peristomal pain, whereas none in the relocation group reported this issue [51].

Another limitation is the relatively small number of studies evaluating minimally invasive approaches to both relocation and direct repair. As these techniques gain traction, however, further exploration of their associated outcomes summarized mainly across various other procedures in reduced recovery time and perioperative risks apply in this context. Upcoming future research should prioritize large-scale, multi-center randomized controlled trials (RCTs) with uniform protocols for follow-up duration, operative technique, PROs, and complication reporting. Such trials could help address the data gaps identified in our analysis and offer more definitive conclusions about the efficacy and safety of each approach in managing parastomal hernia, ultimately supporting more informed evidence-based clinical decision-making and improving patient care.

## Conclusion

This meta-analysis reveals that direct repair and stoma relocation for parastomal hernia offer comparable safety and efficacy, with no significant differences in recurrence, complication rates, or mortality. While stoma relocation involves longer operative times, it does not lead to worse outcomes. Minor variations in short-term complications were noted, but neither approach demonstrated clear superiority.

These findings suggest that repair techniques can be tailored to patient needs and surgical expertise. As minimally invasive options evolve, further research on recovery and perioperative outcomes will support more personalized and effective treatment strategies in parastomal hernia care.

## Supplementary Information

Below is the link to the electronic supplementary material.

Supplementary file1 (DOCX 256 KB)

Supplementary file2 (DOCX 20 KB)

Supplementary file3 (DOCX 13 KB)
