# Supplementary figures and images for: Parastomal Hernia: direct repair versus relocation: is stoma relocation worth the risk? A comparative meta-analysis and systematic review
Source: Updates Surg. 2025 Mar 31;78(1):177–91. doi: 10.1007/s13304-025-02155-8 (PMC12909359; doi:10.1007/s13304-025-02155-8)

**
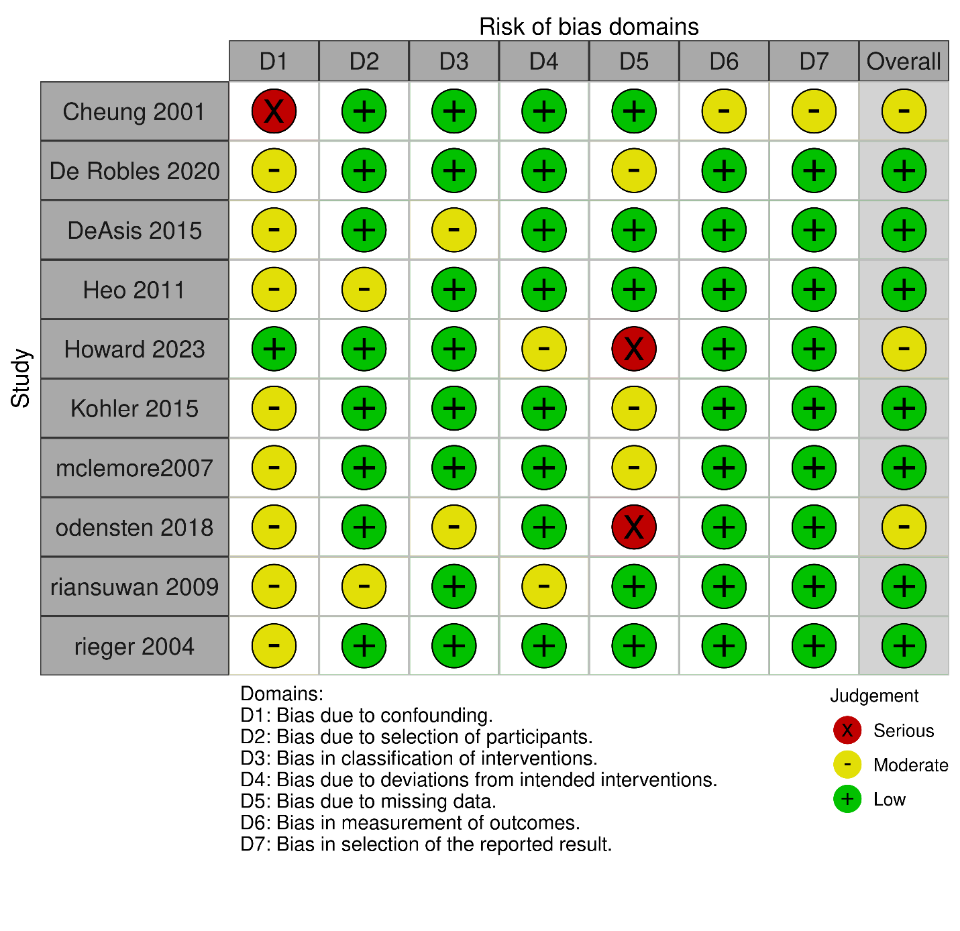
**

Supplementary Table (1): Risk of Bias

Supplement: Supplementary file 1 — Supplementary file1 (DOCX 256 KB) [file 13304_2025_2155_MOESM1_ESM.docx]
