# Supplementary material for: Parastomal Hernia: direct repair versus relocation: is stoma relocation worth the risk? A comparative meta-analysis and systematic review
Source: Updates Surg. 2025 Mar 31;78(1):177–91. doi: 10.1007/s13304-025-02155-8 (PMC12909359; doi:10.1007/s13304-025-02155-8)
Supplement: Supplementary file 2 — Supplementary file2 (DOCX 20 KB) [file 13304_2025_2155_MOESM2_ESM.docx]

The GRADE quality assessment approach indicated that the quality of our evidence-based results is Low to Very low.

Table 1 shows a summary of the evidence's quality, the degree of the effect, and the source of information used in the estimated risk.

**Table 1. The quality of evidence as assessed by GRADE approach**

| **Certainty assessment** | | | | | | | **Certainty** |
| --- | --- | --- | --- | --- | --- | --- | --- |
| **№ of studies** | **Study design** | **Risk of bias** | **Inconsistency** | **Indirectness** | **Imprecision** | **Other considerations** |  |
| **Operative time** | | | | | | | |
| 2 | Retrospective cohort | Not serious | Not serious | Not serious | Very serious^a^ | None | ⨁ Very low |
| **Surgical site infection** | | | | | | | |
| 7 | Retrospective cohort | Not serious | Not serious | Not serious | Not serious | None | ⨁⨁ Low |
| **Urinary tract infection** | | | | | | | |
| 3 | Retrospective cohort | Not serious | Not Serious | Not serious | Serious^b^ | None | ⨁ Very low |
| **Bowel obstruction (ileus)** | | | | | | | |
| 4 | Retrospective cohort | Not serious | Not serious | Not serious | Not Serious | None | ⨁⨁ Low |
| **Length of hospital stay** | | | | | | | |
| 4 | Retrospective cohort | Not serious | Not serious | Not serious | Not Serious | None | ⨁⨁ Low |
| **Overall complications** | | | | | | | |
| 6 | Retrospective cohort | Serious^c^ | Not serious | Not serious | Not serious | None | ⨁ Very low |
| **Readmission** | | | | | | | |
| 3 | Retrospective cohort | Not serious | Not serious | Not serious | Serious^d^ | None | ⨁ Very low |
| **Recurrence** | | | | | | | |
| 7 | Retrospective cohort | Serious^e^ | Not serious | Not serious | Not serious | None | ⨁ Very low |
| **Reoperation** | | | | | | | |
| 4 | Retrospective cohort | Serious^f^ | Not serious | Not serious | Not serious | None | ⨁ Very low |
| **Mortality** | | | | | | | |
| 5 | Retrospective cohort | Not serious | Serious^g^ | Not serious | Not serious | None | ⨁ Very low |

**CI:** confidence interval

#### Explanations

1. Wide CI: 114.66 [95.71, 133.62]
2. Wide CI :1.44 [0.55, 3.78]
3. The proportion of information from studies at high risk of bias(2\6) is sufficient to affect the interpretation of results.
4. Wide CI: 1.05 [0.32,3.44]
5. The proportion of information from studies at high risk of bias (2\7) is sufficient to affect the interpretation of results.
6. The proportion of information from studies at high risk of bias (1/4) is sufficient to affect the interpretation of results.
7. Might represent moderate heterogeneity, I^2^: 41%
