# Supplementary material for: Parastomal Hernia: direct repair versus relocation: is stoma relocation worth the risk? A comparative meta-analysis and systematic review
Source: Updates Surg. 2025 Mar 31;78(1):177–91. doi: 10.1007/s13304-025-02155-8 (PMC12909359; doi:10.1007/s13304-025-02155-8)
Supplement: Supplementary file 3 — Supplementary file3 (DOCX 13 KB) [file 13304_2025_2155_MOESM3_ESM.docx]

|  | **Selection** | | | | **Comparability** | **Outcome** | | | **Quality Score** | **Quality** |
| --- | --- | --- | --- | --- | --- | --- | --- | --- | --- | --- |
| **Study Title** | **Representativeness of the exposed cohort** | **Selection of the non exposed cohort** | **Ascertainment of exposure** | **Demonstration that outcome of interest was not present at start of study** | **Comparability of cohorts on the basis of the design or analysis** | **Assessment of outcome** | **Was follow-up long enough for outcomes to occur** | **Adequacy of follow up of cohorts** |  |  |
| Cheung 2001 | ★ |  |  | ★ |  | ★ | ★ | ★ | 5 | Poor |
| De Robles 2020 |  |  | ★ | ★ | ★ | ★ | ★ | ★ | 6 | Fair |
| DeAsis 2015 | ★ | ★ | ★ | ★ | ★★ | ★ | ★ | ★ | 9 | Good |
| Heo 2011 |  |  | ★ | ★ | ★ | ★ | ★ | ★ | 6 | Fair |
| Howard 2023 | ★ | ★ | ★ | ★ | ★ | ★ | ★ | ★ | 8 | Good |
| Kohler 2015 | ★ |  | ★ | ★ | ★ | ★ | ★ | ★ | 7 | Fair |
| mclemore 2007 |  |  | ★ | ★ | ★ | ★ | ★ | ★ | 6 | Fair |
| odensten 2018 | ★ | ★ | ★ | ★ | ★★ | ★ | ★ | ★ | 9 | Good |
| riansuwan 2009 |  |  | ★ | ★ | ★★ | ★ | ★ | ★ | 7 | Fair |
| rieger 2004 | ★ | ★ | ★ |  |  | ★ | ★ | ★ | 6 | poor |

Table 3. Quality assessment (NOS)
